# Supplementary material for: Reconfigurable nonlinear optical element using tunable couplers and inverse-designed structure
Source: Nanophotonics. 2023 Jun 13;12(14):3019–27. doi: 10.1515/nanoph-2023-0152 (PMC11501354; doi:10.1515/nanoph-2023-0152)
Supplement: Supplementary file 1 — Supplementary Material Details [file j_nanoph-2023-0152_suppl_001.pdf]

Research Article

Vahid Nikkhah, Mario Junior Mencagli, and Nader Engheta*

Supplementary materials: Reconfigurable nonlinear optical element using tunable couplers and inverse-designed structure

1 Topology optimization procedure for the nonlinear element

The distribution of As_2S_3 and Si_3N_4 within the design domain is interpolated by a density variable field $0 \leq \rho(x,y) \leq 1$. $\rho = 1$ is projected to the permittivity of As_2S_3, and the lower bound $\rho = 0$ to the permittivity of Si_3N_4. The intermediate values of the density, which have no physical meaning regarding the wave-matter interaction, must be penalized during the optimization. In other words, the density must be appropriately binarized i.e., at every point, it only takes either zero or one. Also, for controlling the feature sizes of the density distribution and removing small features for future fabrication consideration, a Helmholtz-type spatial filter is applied to the density which filters out domains that are smaller than R_{min}[1]:

$$\rho_{\mathrm{f}}(x,y) - R_{\mathrm{min}}^2 \nabla^2 \rho_{\mathrm{f}}(x,y) = \rho(x,y) \tag{1}$$

where $\rho_{\mathrm{f}}(x,y)$ is the filtered density as the solution to the above differential equation and R_{min} is the filter radius. For penalizing the intermediate values and pushing towards a more binarized distribution, a projection function with a steepness factor p is applied to the filtered density:

$$\rho_{\mathrm{p}} = \frac{1}{1 + e^{-p(\rho_{\mathrm{f}} - 0.5)}} \tag{2}$$

The steepness factor p in the projection function determines the sharpness of the transition of ρ_{f} from 0 to 1 around the middle value $\rho_{\mathrm{f}} = 0.5$. The larger the p parameter is, the more binarized the material distribution would be. During optimization iterations, the steepness factor becomes successively larger. For material distribution, we used a linear interpolation scheme to map the projected density to the permittivity distribution inside the design region as follows:

$$\varepsilon_r(x,y,|E_z|^2) = (1 - \rho_{\mathrm{p}}(x,y))\, \varepsilon_r^{\mathrm{Si_3N_4}} + \rho_{\mathrm{p}}(x,y) \left(\varepsilon_r^{\mathrm{L}} + 3\chi^{(3)} |E_z|^2 \right) \tag{3}$$

Using the adjoint method for calculating the gradient, the optimization algorithm searches through the parameter space spanned by the density variable field to optimize the material distribution to achieve the desired response of the nonlinear power limiter.

For each target input power, P_{in}, we solve the frequency-domain Helmholtz's nonlinear wave equation as:

$$\nabla \times \left(\mu_r^{-1} \nabla \times E_z \right) - \omega_0^2 \mu_0 \varepsilon_0 \varepsilon_r \left(x, y, |E_{\mathrm{z}}|^2 \right) E_z = -j\omega_0 \mu_0 J_z \tag{4}$$

Where E_z is the z component of the electric field throughout the structure, that is excited the current density J_z at the input port boundary. The current density generates an input signal as the fundamental waveguide mode that is injected with an input power P_{in}.

Vahid Nikkhah, Nader Engheta, University of Pennsylvania, Department of Electrical and Systems Engineering, Philadelphia, PA 19104, USA, e-mail: vnikkhah@seas.upenn.edu, engheta@ee.upenn.edu
Mario Junior Mencagli, Department of Electrical and Computer Engineering, University of North Carolina, Charlotte, NC 28223, USA, e-mail: mmencagl@uncc.edu

$\varepsilon_r\left(x, y, |E_z|^2\right)$ is the relative permittivity inside the simulation domain, and it is a function of magnitude of the electric field wherever the Kerr nonlinear material is present. Finally, we set $\mu_0 = 4\pi \times 10^{-7}$ H/m ($\mu_r = 1$) all over the simulation domain. To solve the above equation, we employ the Finite Element Method (FEM) together with Newton-Raphson iterative solver using commercial COMSOL Multiphysics®[2].

For optimization, we set up a cost function to optimize the amount of power reaching Ports 2 and 3 depending on injected power through Port 1. To do so, we define a splitting ratio, which is appropriately defined as a function of the input power, $R(P_{\text{in}})$ as following:

$$\begin{aligned} R(P_{\text{in}}) &= \begin{cases} 1, & P_{\text{in}} \leq P_{\text{sat}} \\ P_{\text{sat}}/P_{\text{in}}, & P_{\text{in}} \geq P_{\text{sat}} \end{cases} \\ P_2 &= R(P_{\text{in}})P_{\text{in}} \\ P_3 &= \left(1 - R(P_{\text{in}})\right)P_{\text{in}} \end{aligned} \tag{5}$$

Where P_2 and P_3 are the signal powers at Ports 2 and 3, respectively. The defined splitting ratio implies that when the input power is below P_{sat}, almost all of it goes to Port 2 and almost none to Port 3. When the input power is above P_{sat} e.g., $P_{\text{in}} = AP_{\text{sat}}$, $A \geq 1$, the power at Port 2 will be $P_2 = RP_{\text{in}} = (1/A)(AP_{\text{sat}}) = P_{\text{sat}}$ satisfying the power saturation at Port 2, and the rest goes to Port 3.

The desired performance is to couple the input signal with power P_{in} to Ports 2 and 3 with the splitting ratio defined in Eq. (5). Therefore, we select N target input powers,P_{in}^k, $k = 1, 2, ..., N$ (shown as the red circles in Fig. 2(b) of the main text), and define the cost function according to:

$$\begin{aligned} L = \sum_{k=1}^{N} \int_{\partial D} \left| E_{\text{z}}^{\text{p2}} - \sqrt{R(P_{\text{in}}^k)P_{\text{in}}^k} E_z^{\text{m,p2}} \right|^2 ds + \\ \int_{\partial D} \left| E_{\text{z}}^{\text{p3}} - \sqrt{\left(1 - R(P_{\text{in}}^k)\right)P_{\text{in}}^k} E_z^{\text{m,p3}} \right|^2 ds \end{aligned} \tag{6}$$

Where E_{z}^{p2} and E_{z}^{p3} are the simulated electric fields at Ports 2 and 3, respectively, at each iteration and corresponding to the target input power P_{in}^k. $E_{\text{z}}^{\text{m,p2}}$ and $E_{\text{z}}^{\text{m,p3}}$ are the electric fields of the fundamental eigenmodes on Ports 2 and 3, respectively, that are normalized with respect to the input power P_{in}^k. ∂D denotes the waveguides' cross-sections covering the waveguides' cores and extends into the air cladding up to a point for covering the electric field profiles of the modes entirely. Minimizing the above cost function gives the desired splitting ratio at target input powers. If the target input powers are close enough to each other, the performance for other values of input powers also becomes close to the desired response.

In the range of input powers for which the element is designed, the local nonlinear response of the Kerr material might be weak. Therefore one would need to enhance the collective power-dependent response of the wave-matter interaction within the design region. There are two options for enhancing the collective power-dependent response. The first is to design the material distribution to create an intense electromagnetic resonance. A strong resonance would be sensitive to a slight change in the nonlinear refractive index due to the varying input power, thus giving us the desired strong collective power-dependent response. However, this approach has the disadvantages of limiting the operational bandwidth and creating large-field hot spots, which would be problematic regarding material damage. Furthermore, a strong resonance is sensitive to any unavoidable imperfection, including nanofabrication errors.

The second option for enhancing the collective intensity-dependent response is to manage for inverse design to achieve a distribution that mostly constitutes the Kerr material. In that case, the EM wave would mostly travel inside the Kerr material to get to the output ports, hence a small modification of the refractive index of the Kerr material along with the long optical path of the wave traveling inside it would enhance the power-dependent response to saturate the power at Port 2. We choose the latter approach for design. One way to stir inverse design to fill most of the design region with Kerr material is simply initializing the density function such that at the start the design region is totally filled with Kerr material i.e., the initial density function is set to $\rho(x, y) = 1$. As the optimization proceeds, the density function at some areas

starts to deviate towards $\rho(x, y) = 0$ (corresponding to Si_3N_4-filled holes) based on the computed gradient and after a few hundred iterations the optimized distribution is achieved.

2 Mathematical characterization of the MZI-based coherent adder

As mentioned in the main text, the MZI-based adder utilized in the proposed nonlinear architecture (Fig. 3(a) of the main text) combines the N complex-valued signals leaving the slope screen into the output signal. To set the MZI phases of the adder, as suggested by Miller we can imagine running it backward by sending a signal from the output port [3–5]. The power injected from the output port needs to be equally divided and to appear at the adder's "input" ports with equal phase. This behavior can be mathematically described by a vector of entries of equal amplitudes and phases, such as [5]:

$$v_a = \frac{1}{\sqrt{N}} [1]_{(N-1)\times 1} \tag{7}$$

Now, we can calculate the MZI phases of the adder, progressively from top to bottom, such that the signals appearing at the "input" ports are equal to v_a [3–5]. Noticing that v_a is a vector of real positive numbers, the common phase of each MZIs (ϕ), which determines the phase of the output signals (see Eq. (2) of the main text), can be set equal to $\pi/2$. The differential phase of the first MZI (the top one) can be derived by equating the first entry of $\mathbf{T}_{\text{MZI}}$ and v_a, which results in $\theta_1 = \arcsin\left(1/\sqrt{N}\right)$. To derive the differential phases of the subsequent MZIs, we need to take into account the propagation of the signal through the previous MZIs. For example, to characterize the second MZI, we can write $\sin\theta_2 \cos\theta_1 = 1/\sqrt{N}$ where the cosine, which comes from the second entry of $\mathbf{T}_{\text{MZI}}$, describes the propagation of the signal through the first MZI. So, the differential phase of the second MZI is given by $\theta_2 = \arcsin\left(\frac{1}{\cos\theta_1\sqrt{N}}\right)$. This process can be generalized to calculate the differential phase of any adder MZIs through the following expression:

$$\theta_k = \arcsin\left(\frac{1}{\sqrt{N}\prod_{i=1}^{k-1}\cos(\theta_i)}\right) \tag{8}$$

with $k = 1, ..., N-1$ and considering the product term equals 1 for $k = 1$. Note that, this characterization of the adder allows conveying all the power carried by the input signals to the output port only if the input signals are equal in amplitude and phase to v_a. In the context of the architecture of Fig. 3(a), as discussed in the main text, the signals have accumulated the same phase before entering the adder, but they have different amplitudes. Thus, the adder correctly adds algebraically the input signals to the output port (with a scale factor of $1/\sqrt{N}$) while not adding all the input power. The remaining power ends up in the bottom-right ports of the adder MZIs.

3 Features of the basis functions

In this section, the features of the basis function in terms of power are discussed. In Fig. S1, the power profiles of the output signals from NSD are plotted with respect to the input power. As expected from the performance of individual power limiters, once the ith output is activated the output power increases linearly until it is saturated at P_{sat} and the spillover goes on to activate the next element. In contrast to the output amplitudes, the output powers comprise a set of piece-wise linear basis over equally-spaced intervals in terms of input power.

In the main text, we demonstrated the approximation of several target nonlinear functions, $f(x)$, by our architecture. We considered the normalized input amplitude with respect to the saturation amplitude as the function's argument i.e., $x = A_{\text{in}}/A_{\text{sat}}$. However, in cases where $f(x)$ varies too fast with x, one might

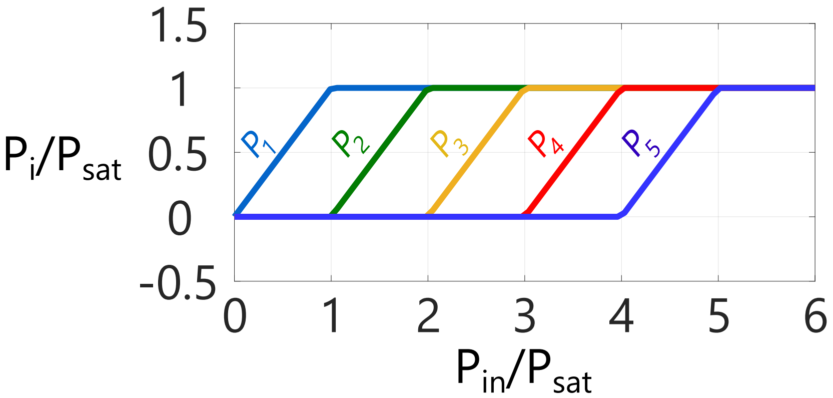

Fig. S1: The power profiles of the NSD outputs vs. the input power.

need to employ more basis for a more accurate approximation. In those cases, one can normalize the input amplitude with respect to a scaled saturation amplitude i.e., $x = A_{\text{in}}/(cA_{\text{sat}}),\quad c > 1$ which contracts the basis intervals. Then by increasing the number of NSD outputs, one can cover the desired domain of the function's argument. For instance, in Fig. S2 the target function from Fig. 4(d) is reconsidered. The blue curve is the plot for $c = 1$ (no basis contraction) and 6 outputs from NSD. One can see the first basis on $0 < x < 1$ cannot accurately follow the target function in this region. In the next scenario, the function's argument domain is contracted by $\sqrt{2}$ and the number of NSD outputs is increased to 12 which results in the red curve approximation. Finally, the green curve is the approximation for $c = 2$ and 24 NSD outputs. As one can see, as the number of NSD outputs is increased one can get a better approximation for a target function which is essentially due to the increased number of sampling points. It is worth noting that the target function here is scaled down by half compared to the target function in Fig. 4(d) for respecting the condition $-1 < s_i < 1$. For this particular example, the optimized slope of the first basis,s_1, gets larger as the contraction factor c increases and to keep $s_1 < 1$ we need to scale down the target function itself.

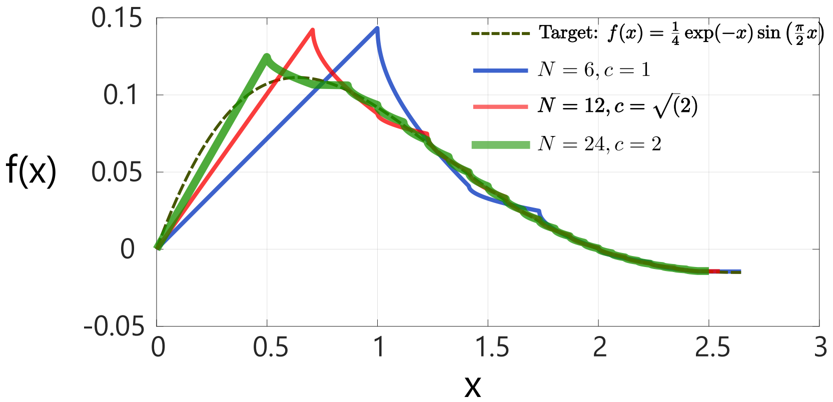

Fig. S2: Approximation the target function $f(x)$ with various x-axis contraction factors c and the number of NSD outputs N.

References

[1] B. S. Lazarov and O. Sigmund, "Filters in topology optimization based on helmholtz-type differential equations," *International Journal for Numerical Methods in Engineering*, vol. 86, no. 6, pp. 765–781, 2011.

[2] "Comsol multiphysics® v. 5.6. www.comsol.com. comsol ab, stockholm, sweden."

[3] D. A. Miller, "Self-configuring universal linear optical component," *Photonics Research*, vol. 1, no. 1, pp. 1–15, 2013.
[4] D. A. Miller, "Self-aligning universal beam coupler," *Optics express*, vol. 21, no. 5, pp. 6360–6370, 2013.
[5] D. A. Miller, "Perfect optics with imperfect components," *Optica*, vol. 2, no. 8, pp. 747–750, 2015.
